# A combination of a ribonucleotide reductase inhibitor and histone deacetylase inhibitors downregulates EGFR and triggers BIM-dependent apoptosis in head and neck cancer

**DOI:** 10.18632/oncotarget.430

**Published:** 2011-01-28

**Authors:** Roland H. Stauber, Shirley K. Knauer, Negusse Habtemichael, Carolin Bier, Britta Unruhe, Simona Weisheit, Stephanie Spange, Frank Nonnenmacher, Verena Fetz, Torsten Ginter, Sigrid Reichardt, Claus Liebmann, Günter Schneider, Oliver H. Krämer

**Affiliations:** ^1^ Molecular and Cellular Oncology/Mainz Screening Center, University Hospital of Mainz, Germany; ^2^ Institute for Molecular Biology, Centre for Medical Biotechnology (ZMB), University Duisburg-Essen, Germany; ^3^ Institute of Biochemistry and Biophysics/Center for Molecular Biomedicine, Friedrich-Schiller-University Jena, Germany; ^4^ Sanofi-Aventis Deutschland GmbH, Industriepark Hoechst, Germany; ^5^ II. Medizinische Klinik, Technische Universität München, Germany

**Keywords:** BH3-only protein, chemotherapy resistance, hydroxyurea, oral cancer, tumor xenograft, valproic acid

## Abstract

Head and neck squamous cell carcinomas (HNSCCs) are the sixth most common malignant neoplasm and more than 50% of patients succumb to this disease. HNSCCs are characterized by therapy resistance, which relies on the overexpression of anti-apoptotic proteins and on the aberrant regulation of the epidermal growth factor receptor (EGFR). As inherent and acquired resistance to therapy counteracts improvement of long-term survival, novel multi-targeting strategies triggering cancer cell death are urgently required. We investigated how induction of replicational stress by the ribonucleotide reductase inhibitor hydroxyurea (HU) combined with histone deacetylase inhibitors (HDACi) exerts anti-tumor activity. We treated HNSCC cell lines and freshly isolated tumor cells with HDACi, such as the clinically approved anti-epileptic drug valproic acid (VPA), in combination with HU. Our data demonstrate that at clinically achievable levels VPA/HU combinations efficiently block proliferation as well as clonogenic survival, and trigger apoptosis of HNSCC cells. In the presence of VPA/HU, such tumor cells increase expression of the pro-apoptotic BCL-2 family protein BIM, independent of wild-type p53 signaling and in the absence of increased expression of the p53 targets PUMA and BAX. The pro-apoptotic activity of BIM in HNSCCs was found critical for tumor cell death; ectopic overexpression of BIM induced HNSCC apoptosis and RNAi-mediated depletion of BIM protected HNSCC cells from VPA/HU. Also, significantly elevated BIM levels (p<0.01) were detectable in the apoptotic tumor centers versus proliferating tumor margins in HNSCC patients (n=31), underlining BIM's clinical relevance. Importantly, VPA/HU treatment additionally reduces expression and cell surface localization of EGFR. Accordingly, in a xenograft mouse model, VPA/HU efficiently blocked tumor growth (P<0.001) correlating with BIM induction and EGFR downregulation. We provide a molecular rationale for the potent anti-cancer activities of this drug combination. Our data suggest its exploitation as a potential strategy for the treatment of HNSCC and other tumor entities characterized by therapy resistance linked to dysregulated EGFR activation.

## INTRODUCTION

With a worldwide annual incidence of more than 640,000 cases, head and neck cancer is the sixth most common malignant neoplasm in humans [1, 2]. The majority of head and neck squamous cell carcinoma (HNSCC) is induced by chronic exposure to a surplus of carcinogens enclosed in all forms of tobacco, synergized by heavy alcohol consumptions and/or is associated with oncogenic human papillomaviruses [3, 4]. HNSCC is characterized by local tumor aggressiveness, high rate of early recurrences and development of second primary carcinomas [3]. Loco-regional relapse after therapy is the major cause of death despite modern disease management strategies [5, 6]. Hence, long-term survival rates, especially for advanced HNSCC (30-40%), have not improved significantly over the last decades [3, 6]. Currently, EGFR-targeting agents, such as antibodies or tyrosine kinase inhibitors gained major clinical attention [3, 7]. Despite encouraging developments, EGFR-directed therapies are effective only in a relatively small percentage of cancer patients underlining the need for additional combination treatment options [7-9].

Therapy resistance favoring recurring or advanced-stage HNSCC mainly results from failure of the tumor cells to undergo chemoradiation-induced apoptosis [1, 3]. Particularly, the intrinsic or mitochondrial pathway of programmed cell death (PCD) plays an important role for killing cancer cells in response to various therapies, and is controlled by interactions among pro- and anti-apoptotic BCL-2 protein family members [10, 11]. Pro-survival proteins like BCL-XL and BCL-2 inhibit apoptosis by binding and neutralizing the activities of the pro-apoptotic multidomain proteins BAX and BAK as well as the BH3 domain-only proteins BIM, BIK, NOXA, and PUMA [10-12].

Overexpression of anti-apoptotic BCL-2 proteins and apoptosis inhibitors like Survivin plays a critical role for therapy resistance and overall survival in HNSCC [10, 11, 13]. Consequently, strategies for neutralizing these cytoprotective factors involve shifting the cellular balance of anti- *versus* pro-apoptotic proteins in favor of the latter [10, 11, 13].

In this respect, histone deacetylase inhibitors (HDACi), such as VPA, have emerged as promising chemotherapeutic agents by inducing a wide range of anti-tumoral activities, including induction of cell cycle arrest and apoptosis [14-20]. HDACi can correct aberrant genomic and non-genomic signaling by chromatin remodeling as well as histone/protein modifications [21]. Likewise, the ribonucleotide reductase inhibitor hydroxyurea (HU) sensitizes tumors to cancer therapy-induced apoptosis and has been used to treat HNSCC, particularly as part of chemoradiation platforms [22, 23]. However, it has not been investigated whether the combination of HDACi and HU may be applicable for the treatment of HNSCC nor have molecular mechanisms underlying its potential anti-tumoral activity been resolved.

Our study demonstrates for the first time that this drug combination efficiently eliminates HNSCC cancer cells by evoking expression of the pro-apoptotic protein BIM (*B cell lymphoma 2 interacting mediator of cell death*) and by downregulation of EGFR. This potent dual anti-tumoral activity suggests the clinical exploitation of this novel drug combination as a strategy to counteract therapy resistance in HNSCC.

## RESULTS

### Combining VPA with HU cooperate in the killing of HNSCC tumor cells and loss of clonogenicity

**Figure 1:**
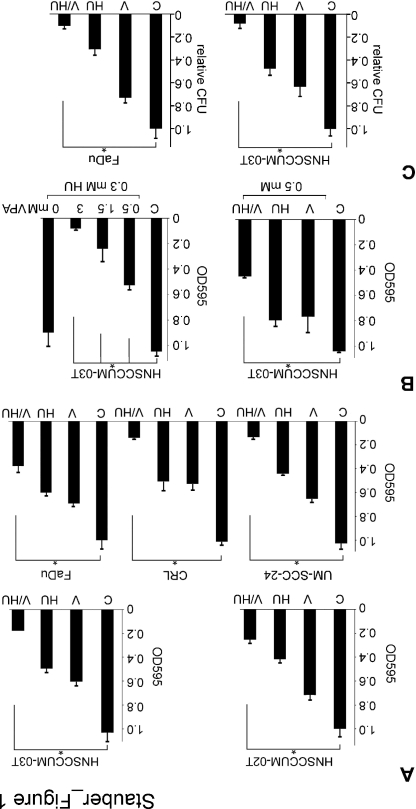
VPA and HU cooperate in HNSCC cells growth inhibition and loss of clonogenicity Columns, mean; bars, ±SD from three independent experiments. A) Indicated cell lines were treated with VPA (V), hydroxyurea (HU), VPA/HU (1.5 mM each) or PBS (C; set as 1) for 48 h, and proliferation was analyzed by the MTT assay. B) Treatment was performed with the indicated drug combinations or PBS (C; set as 1). Cell proliferation was assessed with MTT. C) VPA/HU affects clonogenic cell survival. Cells were seeded and 24 h later treated with the indicated compounds or PBS. Surviving colonies were counted 10 days later and displayed as colony forming units (CFU) relative to the PBS control (C; set as 1)

**Figure 2:**
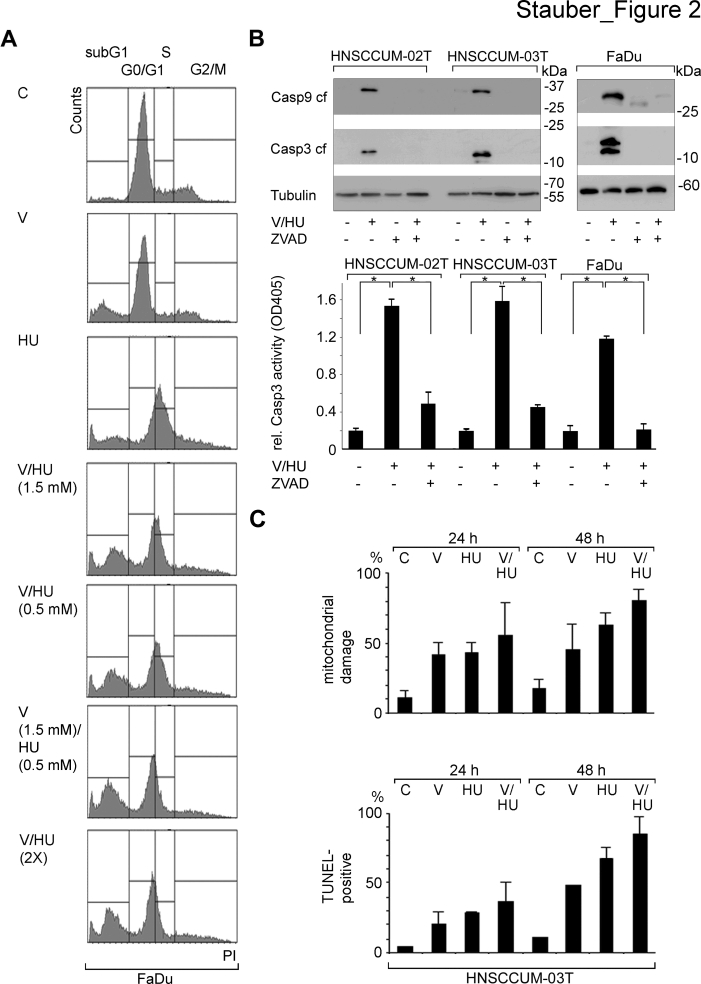
VPA and HU efficiently trigger apoptosis in HNSCC tumor cells A) Drug-induced apoptosis was determined by measuring the sub-G1 population by flow cytometry (PI staining) 48 h post treatment. Induction of cell death was already evident using a single dose of VPA/HU (1.5 mM/0.5 mM) and was not further enhanced by additional drug administration after 24 h (VPA/HU 2X). B) VPA/HU treatment (1.5 mM each; 48 h) induced caspases activation sensitive to the pan-Caspase inhibitor Z-VAD. Immunoblot analysis demonstrated cleavage of Caspase-3 and -9 (upper panel; tubulin, loading control. Apoptosis was quantified by measuring Caspase-3 activity in cell lysates (lower panel). C) VPA/HU-induced cell damage shown by analyzing mitochondrial integrity and by TUNEL-staining. The VPA/HU combination (1.5 mM each) caused significant mitochondrial damage already 24 h post treatment, resulting in loss of dimeric *MitoCapture* dye staining (upper panel). TUNEL-staining revealed VPA/HU-induced DNA-damage indicative of apoptotic cells (lower panel).

Cell lines representing HNSCC from different anatomical sites (Supplementary Table SI) were treated with VPA and HU alone and in combination. MTT assays revealed that although VPA and HU individually inhibited proliferation in a dose-dependent manner, co-administration of VPA/HU was most effective (Figure 1A and B; Supplementary Table SI). Similar results were obtained using a clonogenic cell survival assay (Figure 1C). FACS analysis showed that the VPA/HU combination potently induced apoptosis and confirmed that HU induced S-phase arrest (Figure 2A; Supplementary Figure S1A). Induction of cell death was already evident using a single dosage of VPA/HU (0.5 mM/0.3 mM) and was not dependent on repetitive drug administration (Figure 2A). VPA/HU-induced apoptosis was further confirmed by independent experimental approaches. Immunoblot analysis showed enhanced cleavage of Caspases-3, -8 and -9 (Figure 2B; Supplementary Figure S1B). Also, increased Caspase-3 activity was detectable in lysates from treated cells, which could be counteracted by the pan-Caspase inhibitor Z-VAD-FMK (Figure 2B). The observed cleavage of Caspase-9, the loss of mitochondrial integrity, and DNA fragmentation upon treatment strongly imply that the intrinsic apoptosis pathway is responsible for VPA/HU induced cell death (Figure 2C; Supplementary Figure S1C). Similar results were obtained for several HNSCC cell lines tested and this effect was not restricted to VPA as treatment with other HU/HDACi combinations, such as TSA or butyrate, also resulted in cell death (Supplementary Table SI; Supplementary Figure S1D; data not shown).

### Induction of the pro-apoptotic protein BIM by VPA/HU treatment correlates with cell death

**Figure 3:**
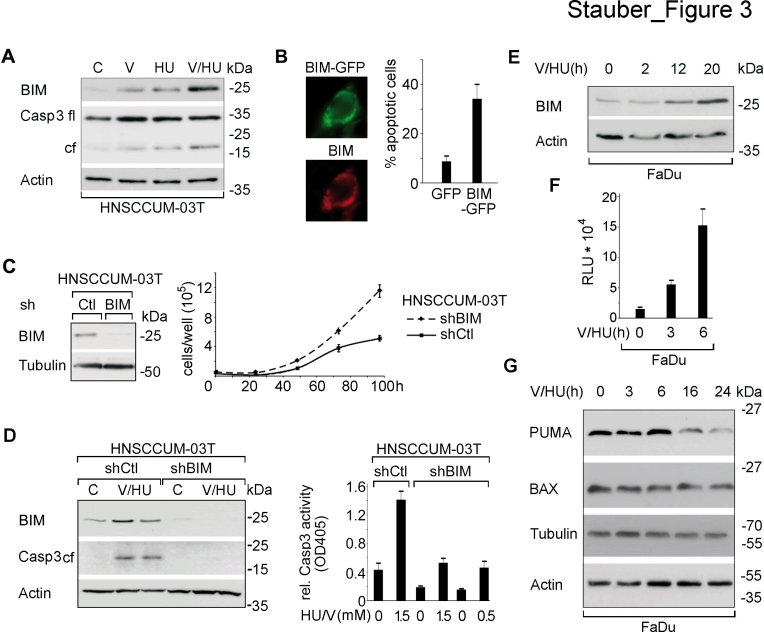
VPA/HU-treatment specifically induces the BCL-2 family protein BIM modulating cell proliferation and apoptosis A) Cells were drug treated (1.5 mM; 20 h) and expression levels of the indicated proteins were visualized by immunoblot. Actin served to control equal loading of cell lysates. B) Cell death induction by ectopic expression of BIM_EL_-GFP. BIM_EL_-GFP was visualized 24 h post transfection in FaDu cells by direct and indirect immunofluorescence using α-BIM Ab. C) Downregulation of BIM in HNSCCUM-03T cells stably transfected with BIM- (shBIM) *versus* scrambled-shRNA (shCtl) verified by immunoblot. Counting revealed that cells with attenuated endogenous BIM levels displayed enhanced proliferation. D) Decreased VPA/HU-induced apoptosis (1.5 mM each, 24 h) in BIM-depleted cells shown by immunoblot analyses for BIM and cleaved Caspase-3 (left), as well as by quantification of enzymatic Caspase-3 activity in cell lysates (right). E) Immunoblot revealed that VPA/HU (1.5 mM each) induced BIM in a time-dependent manner. F) VPA/HU-mediated transcriptional activation was monitored by analyzing luciferase activity. FaDu cells transfected with a BIM reporter were treated with VPA/HU (1.5 mM each). G) In contrast to the strong induction of BIM levels by VPA/HU, correlating with Caspase-3 cleavage, no enhanced expression of PUMA and BAX was induced by VPA/HU. Actin and Tubulin served as loading controls. Columns, mean; bars, ±SD from three independent experiments.

When analyzing the effects of VPA/HU treatment on the levels of pro- and anti-apoptotic BCL-2 proteins, we observed increased BIM levels (Figure 3A). Although both drugs slightly induced expression of BIM, the effect was most prominent using the VPA/HU combination, correlating with enhanced apoptosis. The doses required to induce appreciable Caspase-3 activation and apoptosis were comparable to those necessary to induce BIM expression (Figure 2 and 3A). Notably, this effect was not restricted to VPA as treatment with other HU/HDACi combinations, such as with TSA or butyrate, also resulted in BIM induction and cell death (Supplementary Figure S1D; data not shown). Another BH-3-only protein, p53-upregulated modulator of apoptosis (PUMA), was recently reported to mediate apoptosis induced by EGFR inhibitors in HNSCC cells [12]. In contrast to the strong induction of BIM by VPA/HU, immunoblot analysis revealed no enhanced expression of PUMA, BAX, and BCL-2/BCL-xL (Figure 3G and data not shown). Moreover, VPA/HU could induce BIM in p53-negative PC3 cells, and BIM induction could be verified by independent methods in p53-mutant FaDu cells (Supplementary Figure S1E, S2A).

### BIM is critical for VPA/HU-induced tumor cell apoptosis

To demonstrate that enhancing BIM levels triggers apoptosis in HNSCC cell lines, we first performed ectopic overexpression studies. Transfection of plasmids encoding a BIM_EL_-GFP fusion or untagged BIM_EL_, the longest BIM isoform (196 amino acids), resulted in efficient cell death (Figure 3B and not shown).

In order to further confirm the direct relevance of BIM for HDACi/HU-induced apoptosis, we used RNAi to deplete endogenous BIM. HNSCC cells with RNAi-mediated attenuated BIM expression displayed enhanced proliferation linked to reduced basal apoptosis rates (Figure 3C and data not shown). Furthermore, compared to the scrambled control, these cell lines showed significantly enhanced resistance to VPA/HU-induced cell death, as verified by analyzing Caspase-3 activation, TUNEL-staining and loss of mitochondrial integrity (Figure 3D; Supplementary Figure S2B). Similar results were observed for TSA or butyrate (data not shown). Collectively, these results provide strong evidence that BIM is critical for the HDACi/HU-induced killing of cancer cells.

### VPA/HU enhances BIM expression p53-independently

Increased BIM levels (Figure 3E) could be the result of transcriptional activation [11, 24]. Transfection of a BIM promoter-containing luciferase reporter revealed that VPA/HU indeed stimulated BIM transcription (Figure 3F). Of note, this was observed in cells bearing inactive p53 (FaDu) as well as in p53-negative cells (PC3) (Supplementary Figure S1D and E). Experimental data collected with E2F- or c-JUN/FOS (AP1)-dependent reporter constructs transfected into HNSCC cells suggest a role of AP1 for the VPA/HU-mediated transcriptional activation of BIM (Supplementary Figure S2D). A BIM reporter containing an inactivated MYB-binding site was still responsive to treatment (Supplementary Figure S2C). Although the pharmacological inhibition of ERK signaling was critical for BIM expression in B-RAF/K-RAS mutant lung tumor cells [11], VPA/HU did not affect ERK levels but still evoked enhanced BIM expression in our cell models (Supplementary Figure S2E).

### BIM induction in tumor cells from head and neck carcinoma patients

**Figure 4:**
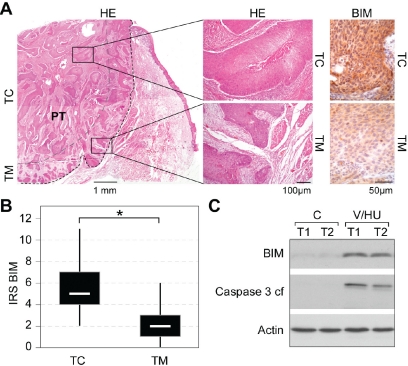
BIM expression in tumor biopsies from head and neck carcinoma patients A) Detection of BIM in HNSCC tumor centers (TC) *versus* proliferating tumor margins (TM). Representative example of an oral SCC (G2, pT3, pN0) stained with hematoxylin/eosin (HE) (left panel) and immunohistochemical visualization of BIM using α-BIM Ab (right panel). B) Box plot (with range) for BIM IRS reveals enhanced BIM expression in the TC in HNSCC patient biopsies (*p<0.001; n=31). C) Treatment of freshly isolated tumor cells from two patients (T1: Hypopharynx, G2, pT3, pN0; T2: oral cavity, G3, pT3, pN0) with VPA/HU (1.5/0.5 mM) for 48 h resulted in BIM induction and Caspase-3 activation. Indicated proteins were detected by immunoblot analysis. Actin served as loading control

To underline the pathophysiological relevance of BIM not only for tumor cell models but also for the clinics, we first visualized BIM expression by IHC in tumor biopsies (n=31). Using the immunoreactive score (IRS) [25], significantly elevated BIM levels (p<0.001) were observed in cancer cells in the apoptotic tumor centers *versus* proliferating tumor margins (Figure 4A and 4B). Second, to definitely demonstrate that BIM is induced by VPA/HU also in primary tumor cells, we used cancer cells freshly isolated from HNSCC patients. Treatment of such tumor cells with VPA/HU resulted in enhanced BIM levels and cancer cell death (Figure 4C). Hence, regulated BIM expression appears to be relevant for disease progression and can be modulated by drug treatment.

### VPA/HU attenuates EGFR expression and signaling

**Figure 5:**
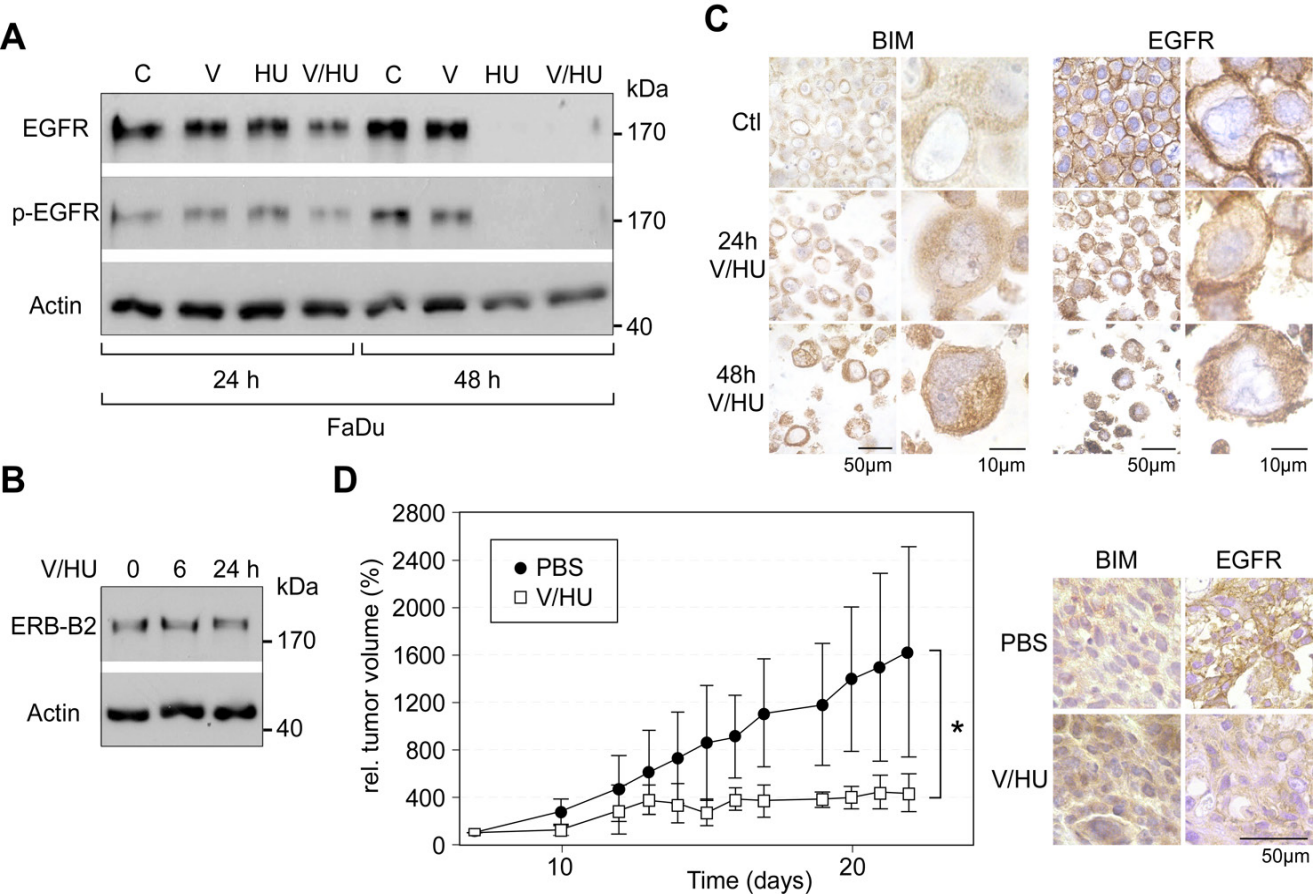
Effects of VPA/HU on the growth factor receptors EGFR and ERB-B2, and suppression of HNSCC tumor growth *in vivo* A/B) FaDu cells were treated with V, HU, VPA/HU (1.5 mM each) or PBS (C). Expression of the indicated proteins was analyzed by immunoblotting. Actin served to control equal loading. VPA/HU treatment effectively reduced the levels of total and phosphorylated EGFR, whereas ERB-B2 levels were not affected. C) FaDu cells treated with VPA/HU (1.5 mM each) were FFPE and used for IHC analysis employing EGFR- or BIM-specific Ab. Treatment resulted in reduced expression and cell surface localization of the EGFR as well as increased BIM levels. D) VPA/HU suppressed the growth of FaDu HNSCC xenograft tumors. Growth curve of tumors subjected to VPA/HU (i.p., 350 mg/kg and 750 mg/kg body weight) or PBS control. Nude mice were inoculated with FaDu tumor cells. When tumors had reached the target size of 0.1 cm^3^, mice were treated once every second day for 14 days. *p<0.001, *n*=4 animals per treatment group, data are mean±SD. E) Enhanced BIM and reduced EGFR levels in xenograft tumors at the end of VPA/HU treatment. BIM and EGFR expression was visualized by IHC.

The EGFR is overexpressed in various epithelial malignancies and also represses BIM expression [11, 26]. As EGFR-targeting strategies are intensively tested in the clinics, we investigated the effects of VPA/HU treatment on this receptor. Interestingly, immunoblot analysis revealed that the combination of VPA/HU efficiently reduced the levels of total and phosphorylated EGFR (Figure 5A). To further examine the intracellular localization of EGFR, cells were treated with VPA/HU or PBS, and examined by IHC analysis. This analysis not only confirmed the reduction of overall EGFR levels, but also showed that such treatment affected the cell surface localization of the receptor and additionally enhanced BIM expression (Figure 5C). As a control, VPA/HU treatment appears not to cause an unspecific degeneration of pro-survival proteins, as STAT3 levels, an important factor for head and neck carcinogenesis [27] or the growth factor receptor ERB-B2, were not significantly affected (Figure 5B; Supplementary Figure S2E). Collectively, these data provide evidence for a hitherto unknown molecular mechanism contributing to the potent anti-cancer activity of the VPA/HU combination. However, we cannot completely exclude the possibility that additional effects of VPA/HU on other proteins may at least partially contribute to the potent anti-tumoral activity of this drug combination.

### VPA/HU efficiently suppresses HNSCC tumor growth in murine xenotransplantation models

Prior to testing the anti-tumoral activity of VPA/HU in murine models, we first compared the cell killing activity of VPA/HU and of chemotherapeutic drugs currently used in the clinics. In our HNSCC cell culture models and at the concentrations used, VPA/HU treatment was more effective in triggering cell death, when compared to the EGFR inhibitors cetuximab and gefitinib or the DNA-damaging agent cisplatin (Supplementary Figure S2F). Notably, interference with EGFR signaling by gefitinib also induced BIM expression (Supplementary Figure S2G).

These *in vitro*-results encouraged us to examine whether VPA/HU treatment also inhibits tumor growth *in vivo*. Using a xenograft model, established FaDu tumors were treated with VPA/HU (350 mg/kg, 750 mg/kg body weight) or PBS control i.p. for 14 days. Administration of VPA/HU to FaDu tumor-bearing mice significantly inhibited tumor growth (p<0.001) (Figure 5D). To visualize whether drug treatment also enhanced BIM levels and caused EGFR attenuation *in vivo*, tumors from treated and control animals were analyzed by IHC. Enhanced BIM levels and reduced EGFR expression were observed in tumors from VPA/HU treated animals compared to those from control mice (Figure 5E). The above data not only confirmed the potent anti-cancer activity of the VPA/HU combination *in vivo*, but also demonstrated the *in vivo*-relevance of the molecular mechanisms identified in our cell culture models. Of note, drug treatment of non-transplanted nude mice did not result in loss of body weight, and no organ damage was evident by histological inspection of treated C57BL/6 mice (data not shown). Hence, the anti-tumoral effect of our drug combination was not mediated by unspecific cytotoxicity.

## DISCUSSION

Rational combination therapies are considered as the most efficient strategy to combat cancer [9, 28]. As the success of these treatments requires a profound molecular knowledge of their underlying mechanisms [29-31], we here studied the combination of two drugs, which have been suggested to affect tumor cell death by different mechanisms [23, 32, 33]. Employing comprehensive cell culture and *in vivo* models we here demonstrate for the first time that combining HDACi with HU potently kills HNSCC by a dual mechanism. Although such agents have been shown to individually affect tumor cells [22, 32], the (pre)clinical anti-tumor activities of the HDACi/HU combinations as well as the underlying molecular mechanisms have not been resolved so far for HNSCC.

Treatment of malignant cells with HDACi can induce a wide range of anticancer effects including apoptosis, cell cycle arrest and differentiation [15, 18, 20, 34, 35]. Numerous HDACi have been tested in the clinics or are currently the subject of ongoing early-phase clinical trials, including HNSCC [14, 19, 36-38]. Since HDACi monotherapies seem not to be effective against solid tumors, their full therapeutic potential will be best realized through combination with other anticancer agents [17, 19, 34]. However, most reports do not provide a well-defined molecular rationale for combining an HDACi with a given agent. Moreover, the molecular events underlying cooperative combination effects are often still to be identified [17, 19, 34].

In contrast, we here provide convincing evidence that activation of the proapoptotic BH3-only protein BIM together with EGFR attenuation are key regulators for VPA/HU-induced tumor cell death. This conclusion is based on several lines of evidence: First, HDACi/HU induced BIM upregulation, induction of apoptosis and loss of the clonogenic potential of HNSCC cell lines derived from different anatomical sites. This finding will be clinically relevant as SCC from different anatomical regions, like the hypopharynx or the oral cavity, differ in their clinical prognosis and response [6], but do express BIM as shown by the analysis of tumor biopsies. Currently, the clinical relevance of results obtained in permanent tumor cell lines are questioned, as such cell lines may differ from primary tumor cells at their molecular level [39]. However, also freshly isolated tumor cells but not fibroblasts from HNSCC patients responded to VPA/HU administered at therapeutically achievable levels (0.5-1.5 mM) with BIM induction, EGFR downregulation and apoptosis. Several clinical studies have already demonstrated that VPA or HU plasma levels in the 0.5-1.5 mM range can be achieved in cancer patients without major toxicity [37, 40, 41], supporting an expectable clinical efficacy of the VPA/HU combination. Second, ectopic expression and RNAi experiments convincingly demonstrate that BIM is the BCL-2 family member essential for VPA/HU-induced cancer cell death. Third, VPA/HU efficiently prevented progression of HNSCC tumors in nude mice correlating with enhanced BIM levels and attenuation of EGFR surface expression. The tumor growth delay achieved with the combination treatment was highly significant compared with the untreated control, without major toxicity.

BIM-evoked apoptosis was found crucial for epithelial tumor cell death triggered by anti-cancer therapeutics [11, 42, 43]. We found that especially the VPA/HU combination cooperatively activated BIM at the transcriptional level. To date, several transcription factors, including p53, E2F, c-JUN/FOS (AP1), MYB, RUNX3, and FOXO3A have been reported to regulate *BIM* transcription [11, 24, 44]. Our data suggest that transcriptional activation of *BIM* in HNSCC by VPA/HU is mediated via AP1, which is not subject to mutation in oral cancer patients [45]. It is possible that HU-mediated S-phase-dependent induction of c-JUN enhances BIM expression [46]. Also, the activity of c-JUN is repressed by the HDAC3-NCoR complex [47], which is particularly sensitive to inhibition by VPA [15]. Phosphorylation of c-JUN by JUN kinase (JNK) permits dissociation of HDAC3 and c-JUN-dependent transcription [47]. HU has been shown to activate JNK *in vivo* [48], which may contribute to c-JUN-dependent *BIM* induction.

Although the BH-3-only protein PUMA was recently reported to mediate apoptosis of HNSCC cell lines induced by EGFR tyrosine kinase inhibitors [12], VPA/HU-mediated PCD did not require induction of the p53 targets PUMA and BAX. This finding seems to be of clinical relevance as VPA/HU-induced cell death does not rely on p53, which is mutated in the majority of HNSCC [49]. Moreover, HNSCC cells with attenuated BIM expression displayed enhanced proliferation. Our finding that lowering this endogenous pro-apoptotic factors not only increases tumor cell survival but also proliferation might be involved in HNSCC therapy response and disease progression. Although BIM plays a major role in death signaling, this does not rule out the additional participation of other BCL-2 family members and/or other apoptosis inhibitor proteins [12, 50].

As we did not observe increased BCL-2/BCL-xL levels upon VPA/HU treatment potentially neutralizing increased BIM expression, it is conceivable to speculate that the addition of BH3 mimetics, such as ABT-737, may not further boost tumor cell death. Killing of *B-RAF* mutant lung tumor cells with ABT-737 required BIM induction by inhibition of ERK signaling. Furthermore, *B-RAF* wild type cancer cells were even largely resistant to this treatment [51]. As the frequency of *RAF/RAS* mutations in HNSCC is rather low [52, 53], VPA/HU is hence likely to be clinically more effective when compared to certain other attempts to alter BCL-2 family members [11]. It will furthermore be interesting to analyze the putative impact of VPA/HU on other molecules important for HNSCC survival. These are for example Aurora kinase-A, which shares signaling pathways with EGFR [54], and the structural protein NSP 5a3a promoting HNSCC apoptosis via the p53-related factor p73 [29, 30].

HNSCC tumors are often characterized by deregulated EGFR signaling due to receptor overexpression, activating receptor mutations and aberrant downstream signaling cascades [7]. Survival is secured by the activation of MEK and ERK kinases, leading to stabilization of MCL-1, activation of BCL-2, and degradation of BIM [11]. (Pre)clinical approaches interfering with EGFR signaling trigger apoptosis by also enhancing BIM expression [7, 11; this study]. Importantly, we show that VPA/HU treatment efficiently reduced not only EGFR levels and signaling, but also attenuated EGFR cell surface localization in cell and xenograft models. Although recent antibody-based EGFR targeting strategies have gained major attention, the clinical response rates to such therapies are rather low. In addition, the mechanism(s) conferring resistance of HNSCC against agents targeting the EGFR are ill-defined [8, 9], and the required agents are expensive and often show a suboptimal pharmacodynamic profile. VPA and HU are stable lower-cost drugs, which can be administered orally [14, 22, 33]. Moreover, the VPA/HU combination may represent a contingency treatment option for patients acquiring resistance to other EGFR-targeting approaches [8, 23, 54, 55]. Both, HU and VPA target major and general cellular survival factors that are misregulated in a variety of human cancers. E.g., cell cycle control is lost in the vast majority of malignancies, and HDACs are frequently overexpressed during tumorigenesis [17]. The molecular mechanisms underlying EGFR attenuation upon exposure of malignant cells to VPA/HU *in vitro* and in mice remain to be resolved in detail. These may involve HU-induced replication arrest, known to affect oncogenic tyrosine kinase signaling [56], and/or the E3 ubiquitin ligase c-CBL, which controls EGFR ubiquitination and lysosomal degradation [57].

A major advantage allowing to expedite potential clinical studies employing VPA/HU for the treatment of HNSCC is the fact that both agents are FDA approved drugs. Albeit hardly in combination, both are used frequently in the clinic [17, 22, 37]. Thus, one can rely on an extensive knowledge on the therapeutically most effective dose and pharmacodynamics of these agents [32, 38]. Also, dose-limiting but clinically manageable side effects are known, including myelosuppression, fatigue, nausea, and diarrhea [22, 32, 33, 37, 38]. In contrast, neither toxicity profiles nor clinical efficacy are known for other anti-tumor strategies targeting BCL-2 family members [51].

Our study has several potential limitations. One is the use of immunocompromised mice as preclinical models of human malignancies, which do not always reflect the heterogeneity and complexity seen in patients. Second, although significantly higher concentrations of VPA or HU were previously used in tumor models without major signs of toxicity [58-60], the VPA/HU doses employed in the xenograft model exceed VPA/HU concentrations currently used in patients [22, 40]. However, cell culture results suggest that even low VPA/HU concentrations will show anti-tumoral efficacy *in vivo*. The minimal effective dose, the optimal application route (oral, i.p., i.v.), as well as the effects of long-term treatment needs to be determined in comprehensive preclinical studies prior to prospective clinical trials.

In conclusion, in addition to current chemo-radiotherapy platforms combining HU with HDACi might prove as an extra treatment option for HNSCC. Although not examined in this study, such drug combinations may be of therapeutic interest also for other tumor entities characterized by therapy resistance and EGFR overexpression, such as colon cancer.

## METHODS

### Ethics Statement

Investigation has been conducted in accordance with the ethical standards and according to the Declaration of Helsinki and according to national and international guidelines and has been approved by the authors' institutional review board.

### Cells, transfections and luciferase assay

Cultivation of the indicated head and neck cancer and other tumor cell lines has been described in detail [25, 61-64] (Supplementary Table SI). Cell lines constitutively expressing shRNA directed against BIM or a scrambled control were generated by transfection of pHR-THT-BIMshRNA-SFFV-eGFP or pHR-THT-scr_shRNA-SFFV-eGFP [65], respectively. Cells were transfected using PEI (Sigma Aldrich, Munich, Germany) or Lipofectamine (Invitrogen, Karlsruhe, Germany) and selected by addition of puromycin (1 μg/ml; Sigma Aldrich, Munich, Germany). Luciferase reporter assays were carried out in triplicate and repeated thrice as stated [62].

### Microscopy and image analysis

Observation, image analysis and quantification of protein localization were performed as described [66]. DNA/cell nuclei were visualized by Hoechst 33258 staining (Sigma Aldrich, Munich, Germany) as described before [66]. At least 100 fluorescent cells were analyzed in three independent experiments.

### Patients, tissue sampling and primary cell isolation

Biopsies of patients diagnosed with HNSCC and treated at the Departments of Oral and Maxillofacial Surgery and ENT of the University Hospitals in Frankfurt and Mainz were analyzed. Tumor specimens were collected from primary tumors of patients who underwent surgery. All cases were clinically and histologically diagnosed according to established criteria including grading and TNM-classification (Supplementary Table SII). Studies of human tissue biopsies were performed according to the requirements of the local ethics committee (#83756604), and informed consent has been obtained in accordance with the Declaration of Helsinki. For the isolation of primary cancer cells, tumor specimens were cut into small pieces and enzymatically digested with collagenase typeI/hyaluronidase (Sigma Aldrich, Munich, Germany) in RPMI-1640 (Invitrogen, Karlsruhe, Germany) at 37°C overnight. Following digestion, dissociated cells were passed through a cell strainer, and epithelial cancer cells and fibroblast were isolated by MACS^®^ separation using CD326 (EpCAM) or α-fibroblast MicroBeads (Miltenyi Biotec GmbH, Bergisch Gladbach, Germany) according to the manufacturer's recommendations. Cells were propagated for one week as described [67] and subjected to analysis.

### Drug treatment and clonogenic survival assay

Cells were treated with VPA, trichostatin A (T), sodium butyrate (B), HU, or cisplatin (Sigma Aldrich, Munich, Germany) as described [16, 67]. The EGFR antagonists gefitinib (Tocris Bioscience, Ellisville, USA) and cetuximab (ImClone, New York, NY, USA) were applied for 48 h. For colony formation assays, 1×10^3^ cells/T25 flask were seeded in triplicate. 24 h later, cells were treated with the indicated compounds or PBS control and further cultivated for 10 days. Drug-containing medium was replaced every day. Cells were fixed and stained with Giemsa. Colonies containing >50 cells were counted automatically using a colony counter (Oxford Optronics, Oxford, United Kingdom). Data shown are calculated from the mean values of three independent experiments.

### Antibodies (Ab)

Ab were: α-PUMA (4976) (NEB Cell Signaling, Frankfurt, Germany); α-Survivin (Novus NB 500-201; Novus Biologicals, Littleton, CO); anti-β-Actin (A2066), α-BIM (B7929), anti-alpha-Tubulin (T5168) (Sigma Aldrich, Munich, Germany); α-BCL-XL (66461A), α-Caspase-8 (9745), -9 (9501) (Pharmingen); cleaved Caspase-3 (9664) (Cell Signaling); α-BAX (sc-20067), α-Caspase-3 (sc-7272/-7148), α-ERB-B2 (sc-284), α-EGFR (sc-81449), α-ERK1/2 (sc-135900), α-STAT3 (sc-482) (Santa Cruz Biotechnology, Heidelberg, Germany). Appropriate HRP-, Cy3- or FITC-conjugated secondary antibodies (Sigma Aldrich, Munich, Germany; Santa Cruz Biotechnology, Heidelberg, Germany) were used.

### Protein extraction, immunoblot analysis and immunofluorescence

Preparation of whole lysates from cells or tissue, co-immunoprecipitations and immunoblotting were carried out as described [61, 62]. Equal loading of lysates was controlled by reprobing blots for Actin or Tubulin as described [62]. Immunofluorescence was performed as described in detail [62, 66, 68].

### Immunohistochemistry (IHC)

Tissue samples or transfected cell pellets were formalin fixed, paraffin embedded (FFPE) and processed for IHC as described [25, 61]. For antigen retrieval, sections were treated in a pressure cooker with Tris buffer (10 mM, pH9.0) for BIM or were treated with proteinase K (S3020, DakoCytomation, Glostrup, Denmark) for 8 min at room temperature for EGFR detection. Sections were incubated with primary Ab (α-BIM, 1:800; α-EGFR 1:50) overnight at 4°C. For visualization, the EnVision^®^ detection system (Dako GmbH, Hamburg, Germany) was applied as described [25]. Sections were counterstained with hematoxylin. Negative control slides without primary Ab were included for each staining. For quantification, sections were scanned at low power to identify areas of positivity and three random fields were selected. Expression levels for BIM were scored semi-quantitatively based on staining intensity and distribution using the immunoreactive score (IRS) [25]. IRS=SI (staining intensity) × PP (percentage of positive cells). SI is assigned as 0, negative; 1, weak; 2, moderate; 3, strong. PP is defined as 0, negative; 1, <5%; 2, 6–30%; 3, 31–60%; and 4, >60% positive cells.

### Measurement of apoptosis, cell cycle and viability

Assessment of apoptosis was performed by quantifying Caspase-3-dependent hydrolysis of a fluorogenic substrate and by immunoblot-based detection of cleaved caspases [62]. Apoptotic cells were visualized by analyzing mitochondrial integrity using the *PromoKine Mitochondrial Apoptosis Staining Kit* (PromoCell; Heidelberg, Germany), staining of fragmented nuclei with Hoechst dye or TUNEL-staining as described [69]. Briefly, 200 cells from three separate images were inspected and the percentage of apoptotic cells determined. Cell viability was calculated employing MTT-assays and the electric sensing zone method as described in detail [61, 62]. Cell cycle profiles were obtained by FACS-mediated analysis of prodidium iodide (PI) stained cells as outlined before [16].

### Animals and xenograft tumors

All animal work has been conducted according to relevant national and international guidelines. For animal studies, FaDu cells (2×10^6^) cells were implanted into both flanks of four-week-old female NMRI *nu*/*nu* mice (Harlan Winkelmann, Hamburg, Germany) [69], and were allowed to establish for 7 days followed by treatment for 14 days. VPA/HU (350 mg/kg, 750 mg/kg body weight) or PBS control was administered intraperitoneally (i.p.) every second day as described [58]. Mice were randomized into groups (4 mice/group) such that the average tumor volumes across the groups were equal. Tumor growth was monitored using calipers to calculate tumor volumes according to the formula: length × π width^2^ × 0.52. Animals were euthanized at the end of the study, and the tumors processed for IHC analysis as described [69]. To assess drug-induced side effects, VPA/HU or PBS treatment was also performed in non-transplanted NMRI *nu*/*nu* or eight-week-old female C57BL/6 mice (2 mice/group). All animal experiments were approved by the Institutional Animal Care and Use Committee at the University of Mainz.

### Statistical analysis

For all experiments stating *p*-values, a paired Student's t-test was performed. Unless stated otherwise, *p*-values represent data obtained from three independent experiments done in triplicate. *p*-values <0.05 were considered as significant.

### Plasmids and RNAi

The expression construct for human BIM_EL_, pCDNA4/TO-BIM_EL_, was described [70]. For expression of a BIM_EL_-GFP fusion, BIM_EL_ cDNA was PCR amplified and cloned into pc3-GFP (pc3BIM_EL_-GFP) as stated before [66]. pGL3-luciferase reporter constructs containing the *BIM* promoter, MYB, E2F or AP1 binding sites were introduced previously [24]. Lentiviral vectors constitutively expressing shRNA directed against BIM or a scrambled control, pHR-THT-BIMshRNA-SFFV-eGFP or pHR-THT-scr_shRNA-SFFV-eGFP, respectively, were reported [65].

## Supplementary Figures and Tables








