# Supplementary material for: A combination of a ribonucleotide reductase inhibitor and histone deacetylase inhibitors downregulates EGFR and triggers BIM-dependent apoptosis in head and neck cancer
Source: Oncotarget. 2011 Jan 28;3(1):31–43. doi: 10.18632/oncotarget.430 (PMC3292890; doi:10.18632/oncotarget.430)
Supplement: Supplementary file 1 [file oncotarget-03-031-s001.docx]

**SUPPLEMENTAL TABLES**

**Supplementary Table SI**

Anatomical origin, EGFR status, VPA/HU-induced BIM expression and apoptosis of cell lines used in the study.

**A**

***HNSCC cell line /***

***anatomical origin***

**EGFR**

**expression**

**VPA/HU-induced**

**BIM expression Apoptosis***

***Naso-/Oropharynx***

*Nasopharynx*

Detroit 562 high + ++

*Tonsils*

*Tongue*

UMB-SCC-864 high + + UMB-SCC-969 high + +

HNSCCUM-02T high + ++ SCC-4 (CRL-1624) high + ++

***Hypo-/Laryngopharynx***

*Hypoharynx*

| FaDu | high | + | + |
| --- | --- | --- | --- |
| *Piriform sinus*  HNSCCUM-03T | high | + | ++ |

*Larynx*

**B**

UM-SCC-5 high + ++ UM-SCC-10B high + ++ UM-SCC-24 high + ++ UM-SCC-33 high + ++

***Cancer cell line /***

***anatomical origin***

**EGFR**

**expression**

**VPA/HU-induced**

**BIM expression Apoptosis***

***Prostate***

*PC3 (*B-Raf WT) high + ++

***Lung***

A549 (B-Raf/K-RAS mut) high + ++

*****Cell viability was calculated employing MTT-assays.
