# Supplementary material for: A combination of a ribonucleotide reductase inhibitor and histone deacetylase inhibitors downregulates EGFR and triggers BIM-dependent apoptosis in head and neck cancer
Source: Oncotarget. 2011 Jan 28;3(1):31–43. doi: 10.18632/oncotarget.430 (PMC3292890; doi:10.18632/oncotarget.430)
Supplement: Supplementary file 2 [file oncotarget-03-031-s002.docx]

**Supplementary Table SII**

Tumor stages and clinical characteristics of 31 HNSCC patients examined by IHC analysis.

*M: male; F: female.*

**A**

***Case***

**Staging pT pN**

**Grading Age Sex**

|  | 1 | T2 | N2 | G2 | 47 | M |
| --- | --- | --- | --- | --- | --- | --- |
|  | 2 | T4 | N2 | G2 | 68 | M |
|  | 3 | T4 | N1 | G2 | 46 | M |
|  | 4 | T1 | N2 | G3 | 57 | M |
|  | 5 | T2 | N3 | G2 | 56 | M |
|  | 6 | T2 | N1 | G3 | 50 | M |
|  | 7 | T2 | N0 | G2 | 58 | M |
|  | 8 | T3 | N1 | G2 | 48 | M |
|  | 9 | T3 | N2a | G2 | 58 | M |
|  | 10 | T3 | N2 | G2 | 56 | M |
|  | 11 | T2 | N3 | G2 | 57 | M |
|  | 12 | T2 | N2 | G3 | 56 | M |
|  | 13 | T4 | N3 | G3 | 56 | M |
|  | 14 | T2 | N2 | G2 | 47 | F |
|  | 15 | T2 | N1 | G2 | 58 | F |
|  | 16 | T3 | N2 | G2 | 58 | F |
|  | 17 | T3 | N2 | G2 | 72 | M |
|  | 18 | T3 | N1 | G2 | 49 | M |
|  | 19 | T4 | N2c | G3 | 66 | M |
|  | 20 | T2 | N2b | G2 | 53 | M |
|  | 21 | T4 | N2 | G2 | 51 | M |
|  | 22 | T3 | N1 | G2 | 47 | M |
|  | 23 | T3 | N2 | G2 | 56 | M |
|  | 24 | T4 | N1 | G2 | 42 | F |
|  | 25 | T4 | N3 | G3 | 56 | M |
|  | 26 | T3 | N2 | G2 | 49 | M |
|  | 27 | T3 | N1 | G2 | 58 | M |
|  | 28 | T1 | N2 | G3 | 57 | M |
|  | 29 | T2 | N3 | G2 | 57 | M |
|  | 30 | T2 | N2 | G2 | 47 | M |
|  | 31 | T4 | N2 | G2 | 80 | M |
| **B** |  |  |  |  |  |  |

**Parameter**

**Anatomical site**

pT1/2 pT3/4

| **Naso-/Oro- pharynx (*N*)** | **Hypo-/Laryngo- pharynx (*N*)** |
| --- | --- |
| 10 | 3 |
| 12 | 6 |
| 5 | 3 |
| 17 | 6 |
| 0 | 0 |
| 18 | 6 |
| 4 | 3 |
| 22 | 9 |

N0/1

N2/3

G1

G2

G3

Σ
