# Supplementary figures and images for: A combination of a ribonucleotide reductase inhibitor and histone deacetylase inhibitors downregulates EGFR and triggers BIM-dependent apoptosis in head and neck cancer
Source: Oncotarget. 2011 Jan 28;3(1):31–43. doi: 10.18632/oncotarget.430 (PMC3292890; doi:10.18632/oncotarget.430)

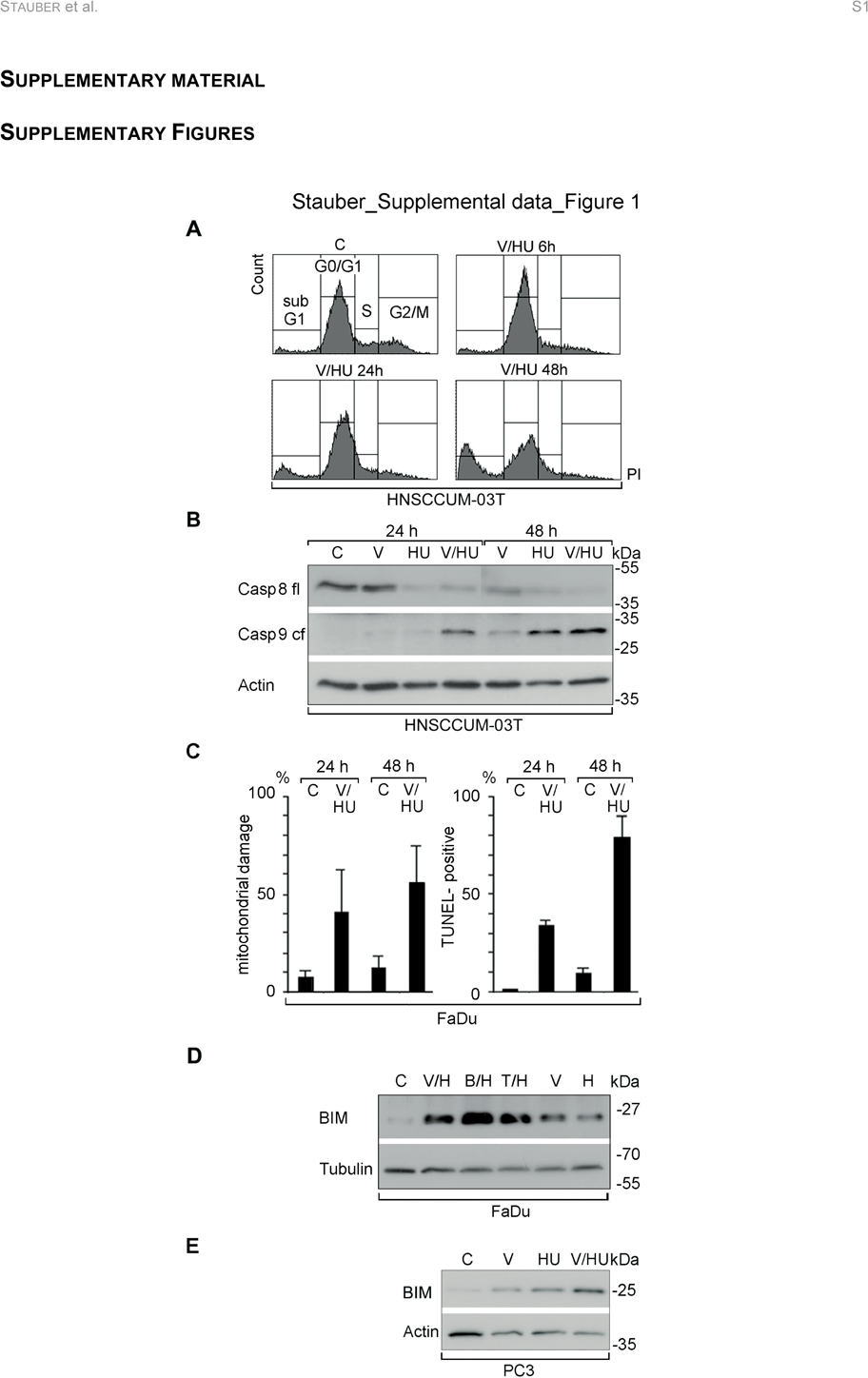

Supplement: Supplementary file 3 [file oncotarget-03-031-s003.tif]

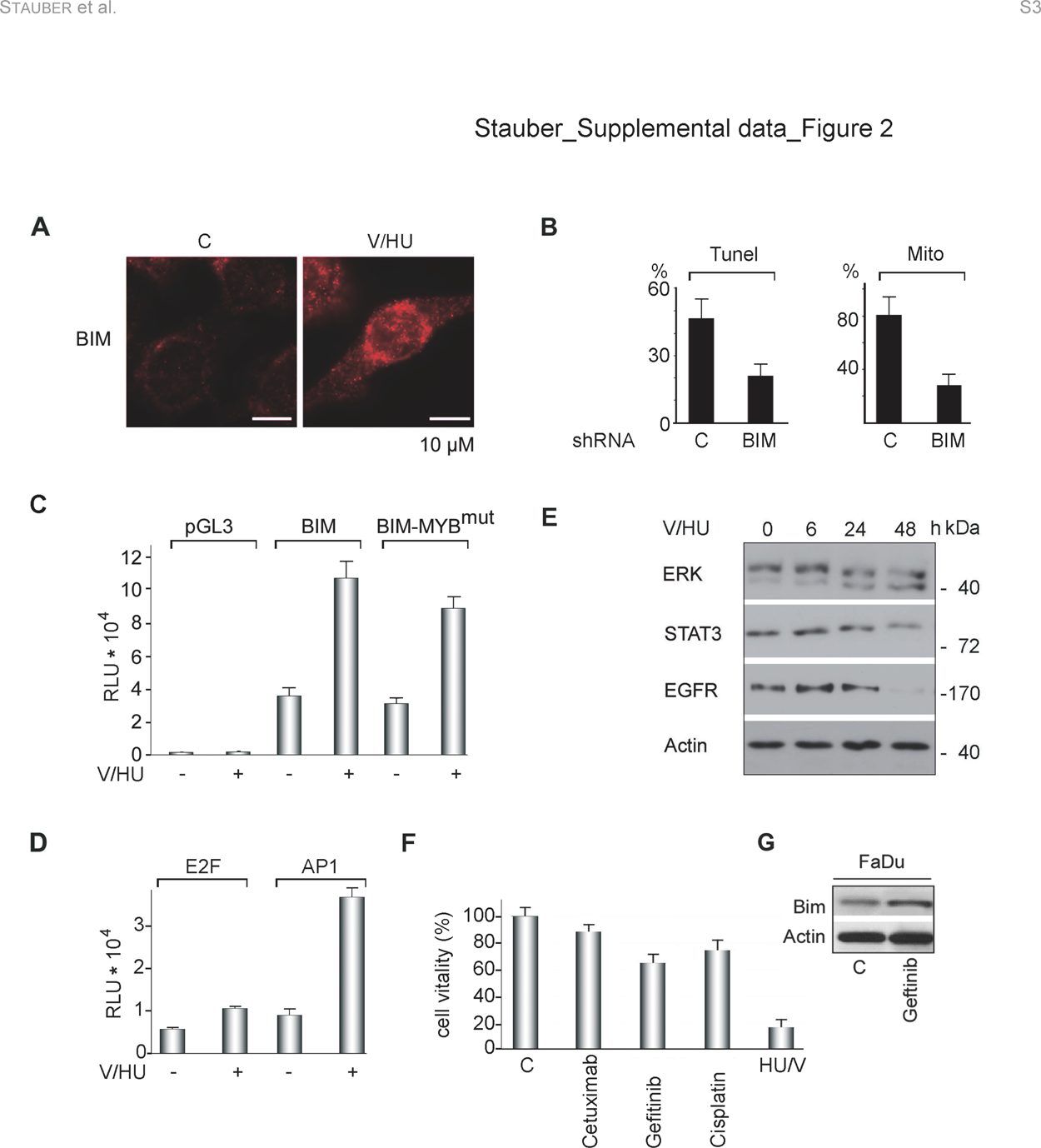

Supplement: Supplementary file 4 [file oncotarget-03-031-s004.tif]
